# Carbon Monoxide Poisoning during Pregnancy: Presentation of a Rare Severe Case with Fetal Bladder Complications

**DOI:** 10.1155/2015/687975

**Published:** 2015-03-05

**Authors:** Myriam Delomenie, Floriane Schneider, Joëlle Beaudet, René Gabriel, Nathalie Bednarek, Olivier Graesslin

**Affiliations:** ^1^Department of Obstetrics and Gynecology, Hôpital Maison Blanche CHU Reims, 51100 Reims, France; ^2^Department of Pediatrics, Hôpital Maison Blanche CHU Reims, 51100 Reims, France

## Abstract

Carbon monoxide poisoning during pregnancy is a rare and potentially serious condition. Fetal complications are uncommon, related to anoxic lesions. The severity of these complications does not depend on the level of maternal COHb. We report the case of a 22-year-old pregnant woman who at 30 weeks of gestation had carbon monoxide poisoning secondary to a fire in her home, complicated by cardiac arrest and severe fetal damage. The child had not brain damage, but presented bladder lesions not previously described, with urinary ascites complicating megacystis.

## 1. Introduction

Carbon monoxide (CO) poisoning is relatively rare during pregnancy. It may have potentially serious adverse effects on mother and fetus; in particular, the resulting intrauterine hypoxia can cause fetal death or severe neurological sequelae. We report here the case of a 22-year-old woman who at 30 weeks of gestation had carbon monoxide poisoning. The fetus had not only brain damage but also bladder lesions not previously described, with urinary ascites complicating megacystis.

## 2. Case Report

This 22-year-old pregnant woman was found unconscious in cardiorespiratory arrest following a fire at her home at 30 weeks of gestation. She required external cardiac massage and administration of hydroxocobalamin (Cyanokit). The patient was ventilated for 24 hours. Her pregnancy had been unremarkable to that point.

**Figure 1:**
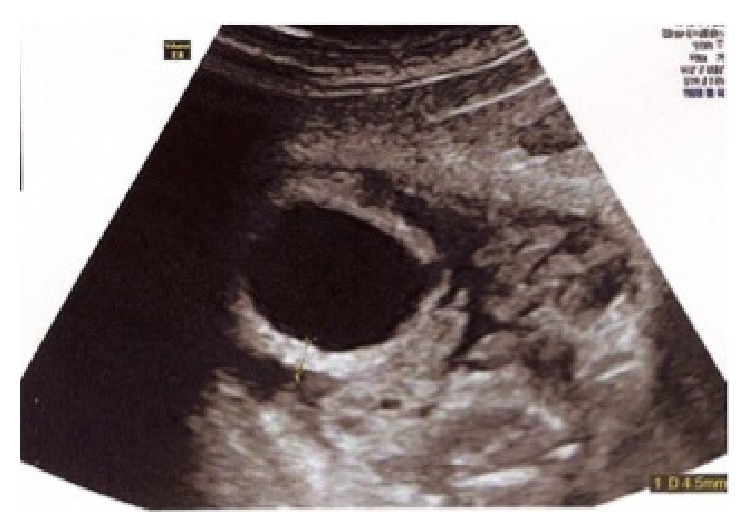
Rupture of detrusor and hypertrophic bladder.

Initial maternal COHb was measured at 52%, pH = 7.47, PaO_2_ = 71 mmHg, PaCO_2_ = 30 mmHg, Bicarbonates = 21.6 mmol/L, BE = −1, and FiO_2_ = 50%. After one 90-minute hyperbaric session, COHb fell to 6.6%. Obstetric ultrasound in the intensive care unit at admission found an eutrophic fetus with a normal amount of amniotic fluid and no obvious morphological abnormalities (bladder of normal size, normal central nervous system). The fetal heart rate was tachycarding then micro oscillating and unresponsive. At 30.5 weeks, the bladder volume had increased sharply (67 × 40 mm) with normal-sized kidneys. At 31.5 weeks, megacystis was still visible, and ascites appeared. The ultrasound scan a few days later showed a ruptured bladder detrusor muscle (Figure 1), moderate bilateral hydronephrosis, and a normal amniotic fluid index.

**Figure 2:**
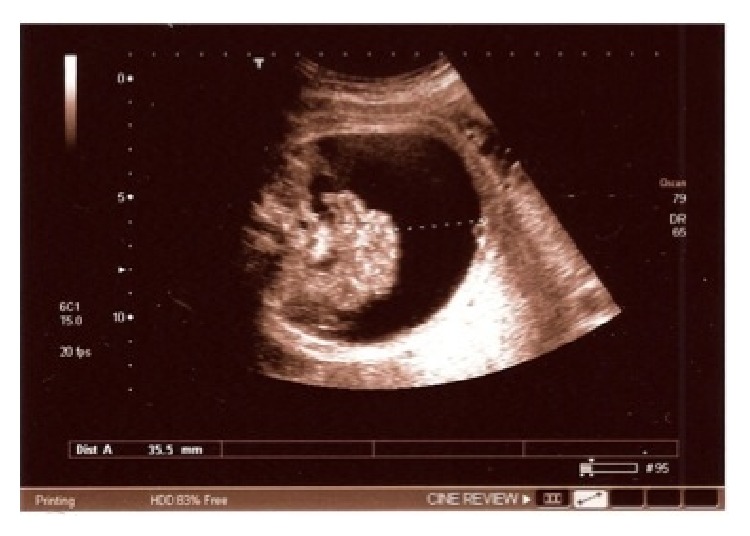
Urinary ascites.

At 32.5 weeks, major fetal urinary ascites appeared, bladder size decreased, and oligoamnios was visible (Figure 2).

**Figure 3:**
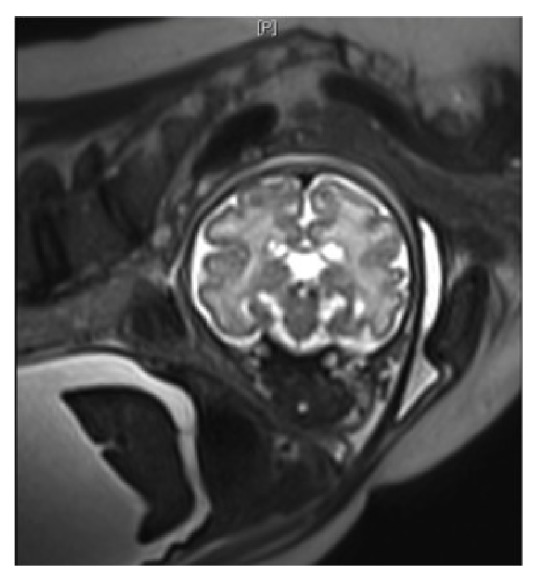
Cephalic antenatal fetal MRI. Coronal T2. Cysts of the caudate nuclei.

Fetal brain MRI was performed at 33 weeks. Hypointense signals were visible on T1-weighted images and hyperintense signals on T2, both bilateral, at the posterior limb of the internal capsule; they corresponded to signs of asphyxia. Cortical involvement was observed, with laminar necrosis detected by T2 hypointense cerebral cortex signals. Cysts in the caudate nuclei corresponded to areas of periventricular leukomalacia (Figure 3). Because of the very negative prognosis, a pregnancy termination was discussed with the parents.

At 34 weeks, the ultrasound images showed that the size of the bladder was normal, as was the appearance of the kidneys.

A 2400-gram baby, Chloé, was delivered at 35.5 weeks. Urination was spontaneous at birth, pH 7.30, PaCO_2_ = 56.7 mmHg, HCO_3_^−^ 27.4 mmol/L, and COHb 2.4%. Seizures and hypotonia were observed.

Abdominal ultrasound found an enlarged bladder without ascites.

**Figure 4:**
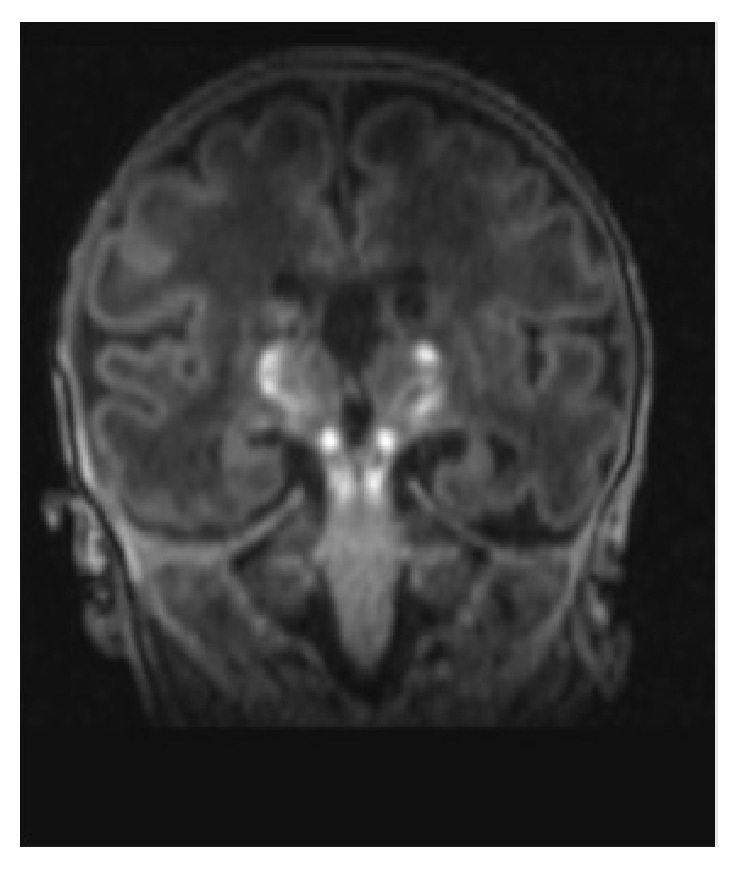
Postnatal cephalic MRI. T1 hyperintense pyramidal tract lesions.

Postnatal cerebral MRI revealed heterogeneous bilateral and symmetric T2 signals in the central grey nuclei, consistent with the anoxic-ischemic lesions formed, mild ventricular dilatation due to a defect, poor differentiation of the cortex lesions, and T1 hyperintensity of the pyramidal tract (Figure 4).

Chloé presented spastic quadriparesis with spontaneous breathing but hypoventilation. She could not suck, and she drooled while swallowing. She died at 2.5 months of life from respiratory distress.

## 3. Discussion

Monoxide carbon exposure during pregnancy can cause severe damage, including intrauterine hypoxia, serious neurological damage, and even fetal death. CO dissolved in maternal plasma crosses the placenta by passive diffusion and thus combines with fetal haemoglobin. The fetal COHb levels created are 10–15% higher than maternal levels, as fetal haemoglobin has a higher affinity for CO than adult haemoglobin. Moreover, fetal elimination of CO takes more time, for it dissociates much more slowly from fetal than adult haemoglobin [1]. For this reason, the severity of fetal intoxication cannot be assessed solely by maternal condition.

The only possible nonteratogenic treatment for pregnant women with CO poisoning is hyperbaric oxygen therapy [2–4], as shown by Elkharrat et al. [5]. Hyperbaric oxygen is mandatory for all pregnant women with either impaired consciousness or COHb levels of 20% or higher [6]. The literature shows that COHb > 48% is most often, but not always, associated with a poor prognosis [7].

The effects of carbon monoxide on fetal development differ by term. During the embryonic phase, CO can cause a variety of birth defects. During the fetal stage, congenital anomalies are less common, but death or permanent neurological damage may occur [8]. Studies of monkeys and of cats after inhalation of carbon monoxide found that the most vulnerable areas of the brain are those containing white matter (periventricular, especially) and the brainstem, followed by the thalamus and the cerebral cortex [9].

The child in this case had cerebral complications linked both to the anoxia-ischemia following the CO poisoning, as explained above, and to the low blood flow due to the mother's cardiorespiratory arrest. The ensuing lesions of the pyramidal tracts (anterior and posterior limbs of the internal capsule to the brain stem) resulted in spastic quadriparesis and swallowing disorders. Behavioural disorders can also be explained by the diffuse cortical involvement.

The novelty of this case lies in the development of megacystis, which was complicated by rupture and ascites and then resolved spontaneously and almost completely at birth. No case in the literature describes this type of complication. The fetal ultrasound examinations performed before the fire did not show any digestive abnormalities or related findings.

The outcome was self-limited, with regression of urinary ascites and spontaneous urination at birth. Ultrasound at birth found an enlarged bladder but no associated anomalies. The spinal MRI found no injury.

The CO poisoning in this case involving a fire was combined with cyanide poisoning. No cases of fetal cyanide poisoning have been reported in the literature.

No reports of the use of hydroxocobalamin in pregnant women describe any teratogenic effects [8], nor have we found any case describing bladder lesions secondary to fetal hypoxia in the context of cardiac arrest.

Finally, descriptions of idiopathic megacystis are extremely rare in the literature.

The mechanism of this neurogenic bladder may be linked to the lesions of the central grey nuclei (i.e., the basal ganglia): the neurological shock might have blocked bladder motility, especially as supraspinal structures, including the basal ganglia and cortex via the pyramidal tract, control the bladder and sphincter reflexes [10]. Specifically, this damage may be due to the significant hypoxia caused by maternal cardiorespiratory arrest from carbon monoxide poisoning. But fire smoke can contain several poisons, depending on the type of the burned substance and ambient oxygen concentration. The most notable combustion products are CO, cyanide, and irritant gases (e.g., phosgene, acrolein, chlorine, and ammonia). All of them can cause hypoxic damage via different mechanisms; a synergistic effect is probable.

Other mechanisms for the bladder injury such as low cyanide detoxification due to small bladder content of rhodanese (thiosulfate: cyanide sulfurutransferase), CO induced smooth muscle relaxation, and hypoxic induced diabetes insipidus should be discussed.

The seriousness of carbon monoxide poisoning in pregnancy is due to the vulnerability of the fetus and the difficulty of ascertaining the extent of its poisoning. Accordingly, hyperbaric oxygen therapy is urgent as soon as the mother presents signs of acute CO poisoning [1].
